# Evaluation of Anti-*Toxoplasma gondii* Effect of Ursolic Acid as a Novel Toxoplasmosis Inhibitor

**DOI:** 10.3390/ph11020043

**Published:** 2018-05-09

**Authors:** Won Hyung Choi, In Ah Lee

**Affiliations:** 1 Marine Bio Research & Education Center, Kunsan National University, 558 Daehak-ro, Gunsan-si, Jeollabuk-do 54150, Korea; 2 Department of Chemistry, College of Natural Science, Kunsan National University, 558 Daehak-ro, Gunsan-si, Jeollabuk-do 54150, Korea; leeinah@kunsan.ac.kr

**Keywords:** zoonosis, parasites, *Toxoplasma gondii*, infectious disease

## Abstract

This study was carried out to evaluate the anti-parasitic effect of ursolic acid against *Toxoplasma gondii* (*T. gondii*) that induces toxoplasmosis, particularly in humans. The anti-parasitic effects of ursolic acid against *T. gondii*-infected cells and *T. gondii* were evaluated through different specific assays, including immunofluorescence staining and animal testing. Ursolic acid effectively inhibited the proliferation of *T. gondii* when compared with sulfadiazine, and consistently induced anti-*T. gondii* activity/effect. In particular, the formation of parasitophorous vacuole membrane (PVM) in host cells was markedly decreased after treating ursolic acid, which was effectively suppressed. Moreover, the survival rate of *T. gondii* was strongly inhibited in *T. gondii* group treated with ursolic acid, and then 50% inhibitory concentration (IC_50_) against *T. gondii* was measured as 94.62 μg/mL. The *T. gondii*-infected mice treated with ursolic acid indicated the same survival rates and activity as the normal group. These results demonstrate that ursolic acid causes anti-*T. gondii* action and effect by strongly blocking the proliferation of *T. gondii* through the direct and the selective *T. gondii*-inhibitory ability as well as increases the survival of *T. gondii*-infected mice. This study shows that ursolic acid has the potential to be used as a promising anti-*T. gondii* candidate substance for developing effective anti-parasitic drugs.

## 1. Introduction 

Toxoplasmosis is caused in all age groups, including young children or adults globally, which is one of parasitic diseases as zoonosis infected through *Toxoplasma gondii* (*T. gondii*). *T. gondii* not only induces chronic infection in various sites of human body, but it also causes brain infection through the central nervous system as zoonotic parasitosis. In addition, *T. gondii* has unique infectious subcellular organelles, including apicoplast, conoid, rhoptries, dense granules, and micronemes [1]. In general, *T. gondii* forms a unique proliferative membrane, such as a parasitophorous vacuole membrane (PVM) including reticular network (RN) during its proliferation after invasion into host cells, and grows in it [2]. Until recently, several drugs were developed to inhibit *T. gondii*, and they are being used to treat toxoplasmosis’ patients in the clinic. However, their side effects are still clear in the clinic, and it is also being exposed to drug-resistance gradually [3,4,5]. In these aspects, pyrimethamine used for treating toxoplasmosis blocks the synthesis of tetrahydrofolic acid from dihydrofolate reductase (DHFR) by effectively inhibiting the dihydrofolate reductase in *T. gondii*, which inhibits consistently synthesis of the DNA and/or RNA in the proliferation of protozoa species, including malaria. 

Until recently, it was reported that various extracts and/or natural products derived from medicinal plants had the possibility of useful medical resources for treating infectious virus diseases, including middle east respiratory syndrome (MERS), dengue fever or Zika fever, as well as showed in vitro inhibitory effects against chronic diseases, such as hepatitis, cancer, and tuberculosis [6,7,8,9,10,11]. Although various extracts derived from traditional medicinal plants and its natural compounds were reported to indicate anti-*T. gondii* activities/effects [12,13,14,15,16], an effective drug of the next generation for the treatment of toxoplasmosis caused by *T. gondii* has not yet been developed through the clinical study. Furthermore, despite various efforts for developing anti-parasitic drugs, zoonotic parasitosis derived from parasites has been consistently worsening a crisis of the public health worldwide.

In this aspect, various researches for discovering effective drugs against toxoplasmosis, and studies on new substances of relatively low toxicity with safety are urgently required to inhibit zoonosis. Ursolic acid is a bioactive substance contained in various medicinal plants that are used as natural resources in oriental medicine and in folk medicine, and is also known to have a variety of effects and bioactivity, such as anxiolytic activity [17], anti-angiogenic activity [18], and antiepileptic effect [19]. Furthermore, ursolic acid effectively induces extensive bioactivities, including anti-inflammatory [20,21], anticancer [22,23,24], antioxidant [25], antimicrobial [26], and anti-tubercular effects [27], as well as causes strong inhibitory effects against arthritic [28] and autoimmune disease [29]. These studies show that ursolic acid not only induces various physiological activities in both “in vitro” and “in vivo”, but also has the possibility as an anti-parasitic candidate drug. However, studies regarding anti-*T. gondii* activity of ursolic acid has not been reported yet. In addition, a novel and/or effective drug for the treatment of toxoplasmosis has not been developed yet, even though significant results regarding anti-*T. gondii* activity have been reported through various studies globally [30,31]. For this reason, this study started from the hypothesis that ursolic acid may effectively inhibit or modulate the proliferation/growth of *T. gondii,* causing toxoplasmosis in human. This study was carried out to evaluate the anti-*T. gondii* effect of ursolic acid which is known as a bioactive substance, and to determine its potential as a promising candidate substance for developing novel anti-toxoplasmosis drugs. 

## 2. Results 

### 2.1. Effect of Ursolic Acid on the Proliferation and Growth of T. gondii

**Figure 1:**
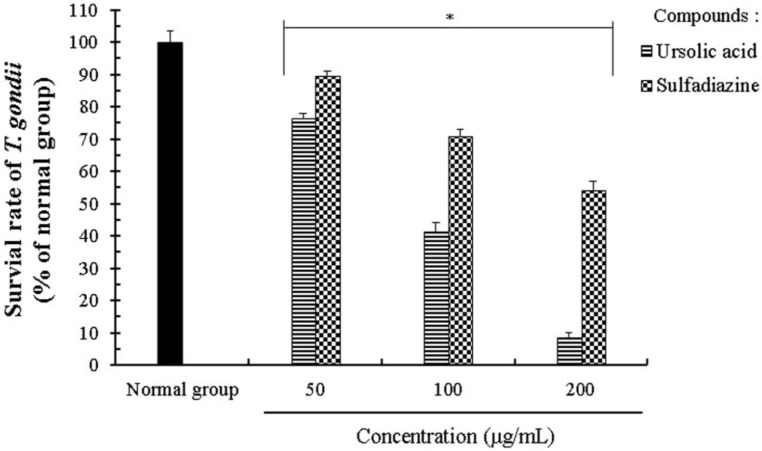
The *T. gondii*-inhibitory effect of ursolic acid regarding *T. gondii*. *T. gondii* was incubated with different concentrations (50–200 μg/mL) of ursolic acid and sulfadiazine for 24 h, respectively. The results were expressed as a percentage of the control group, and all of the results were presented as mean ± standard deviation (S.D.) of three independent experiments. * *p* < 0.05 was considered to be statistically significant.

*T. gondii* causes parasitic disease on both animals and human, as well as having unique micro network systems and a specific structure, including various micro-organelles, such as mitochondria, rhoptries, and micronemes. We evaluated the anti-parasitic activity and the effects of UA on the proliferation and growth of *T. gondii* using an MTT assay and UA showed the anti-*T. gondii* activity in the range of 25–200 μg/mL. *T. gondii*-infected cells were incubated with different concentrations (25–200 μg/mL) of UA for 24 h, and their viability was markedly decreased in a dose dependent manner (Table 1 and Table 2). In addition, the UA effectively inhibited the proliferation and growth of *T. gondii*-infected cells as compared with SF. In particular, *T. gondi*-infected cells treated with UA (100 μg/mL) showed a significant decrease of *T. gondii* including *T. gondii* fragmentation as well as morphological changes such as cell shrinkage and cell fragmentation when compared with the untreated *T. gondii*-infected cells (Data not shown), which suggests that UA has anti-parasitic activity and the inhibitory effect against *T. gondi* in infected cells (Table 2). Furthermore, the UA effectively inhibited the viability of the parasite through the direct inhibition of *T. gondii* when compared with SF, which is to strongly demonstrate its selective inhibitory effect against *T. gondii*, and then the parasitic SR was measured at less than 45% at concentrations of 100 μg/mL (Figure 1). The 50% inhibitory concentration (IC_50_) value of UA against *T. gondii* and *T. gondii*-infected cells was measured as 94.62 μg/mL and 162.25 μg/mL, respectively. These results demonstrate that UA strongly induced anti-proliferation activity of *T. gondii* in the infected host cells by effectively blocking *T. gondii* as well as the direct inhibitory action against *T. gondii*.

### 2.2. Anti-Parasitic Effect of Ursolic Acid on PVM Formed by T. gondii Proliferation 

**Figure 2:**
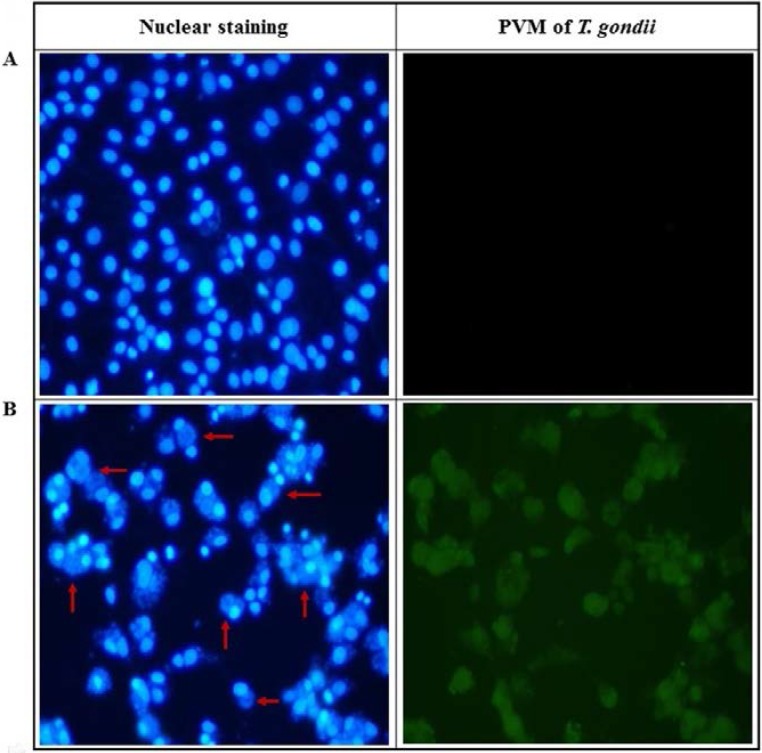

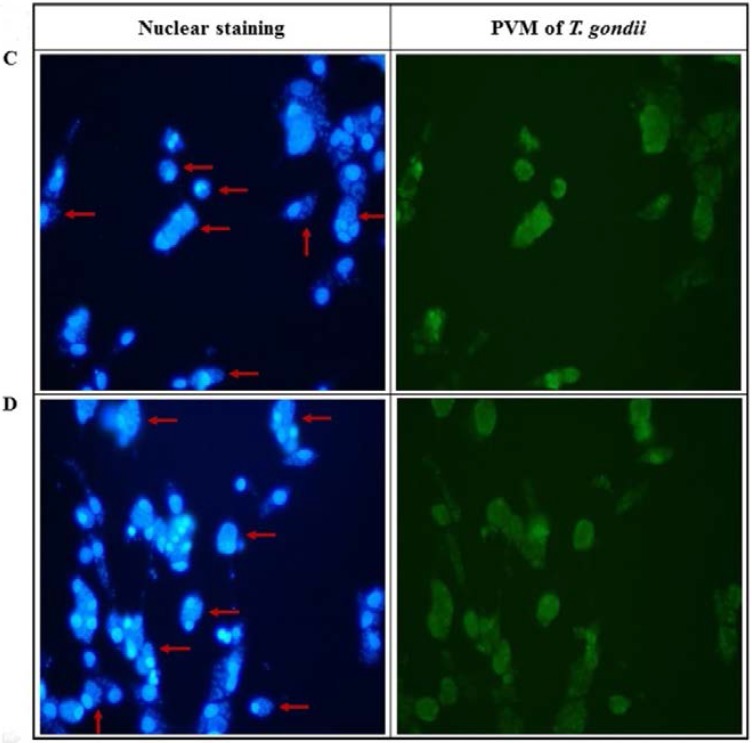
The changes of immunofluorescence of parasitophorous vacuole membrane (PVM) formed in *T. gondii*-infected cells and changes of nuclear staining. (**A**) Uninfected normal cells. (**B**) *T. gondii*-infected cells. (**C**) *T. gondii*-infected cells treated with 100 µg/mL of ursolic acid. (**D**) *T. gondii*-infected cells treated with 100 µg/mL of sulfadiazine. The cells were infected with *T. gondii* (host cells:*T. gondii* = 1:5), and *T. gondii*-infected cells were incubated with the compounds for 24 h. Green fluorescence shows morphology of PVM formed by *T. gondii* proliferation in host cells after *T. gondii* invasion. Blue fluorescence shows nucleus of *T. gondii* group proliferated in PVM, and nucleus of the host cells, respectively (the arrow shows the nucleus of *T. gondii* in PVM formed by *T. gondii* in the host cells).

*T. gondii* induces anti-apoptotic steps and features through the inactivation of apoptotic proteins during its proliferative phase in host cells, which shows the unique parasitic life-cycle of *T. gondii.* In particular, the formation of PVM is accelerated in a time-dependent manner after cell invasion of *T. gondii*. For this reason, we investigated the inhibition of PVM that is caused by the interaction between *T. gondii* and UA during the proliferation stage of *T. gondii* in host cells. As shown in Figure 2, the PVM formation and nucleus of *T. gondii* were markedly decreased in *T. gondii*-infected host cells when it was treated with 100 μg/mL of UA and SF, respectively, and their changes were clearly observed under a UV fluorescence. The results indicate the inhibitory effect of UA against PVM formation and the viability of *T. gondii.* Therefore, these results show substantial evidence that UA effectively inhibited or blocked the PVM formed by *T. gondii* during the proliferative stage as well as the proliferation of *T. gondii* in the infected host cells after cell invasion. 

### 2.3. Effect of Ursolic Acid through the Inhibition of T. gondii in T. gondii-Infected Mice 

**Figure 3:**
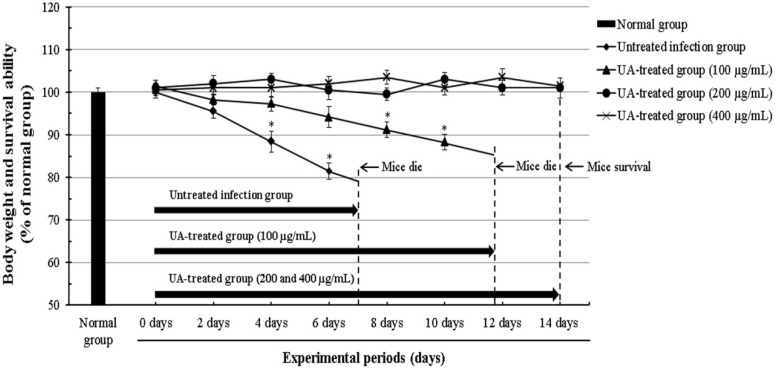
Changes of the body weight and the survival ability in *T. gondii*-infected mice. The mice were divided into normal group (*n* = 5), positive group (*T. gondii*-infected group, *n* = 5) and experimental groups (3 groups of UA, *n* = 15). The mice were infected with *T. gondii* treated by 100, 200, and 400 μg/mL of ursolic acid (UA) through the abdominal cavity of each mouse respectively. The mice were carefully observed during the experiment periods after *T. gondii* infection. The results were expressed as a percentage of the normal group, and presented as the mean ± S.D. * *p* < 0.05 was considered to be statistically significant.

*T. gondii* induces infectious symptoms and diseases, such as lymphadenopathy and brain injury, particularly in human as a zoonotic parasite that is infected in both human and animals. In this aspect, we evaluated the anti-proliferative effect and the parasitic inhibitory activity of UA against the viability of *T. gondii* in *T. gondii*-infected mice. The mice were carefully observed during the experimental periods after the injection of *T. gondii* treated with 100, 200, and 400 μg/mL of UA for 24 h. As mentioned above, *T. gondii*-infected mice treated with different concentrations (200 and 400 μg/mL) of UA showed higher activity and viability than *T. gondii*-infected mice, and the mice indicated the dynamic and energetic activity as the normal group. In particular, it clearly confirmed a significant difference of the survival rate between *T. gondii*-infected mice and the infected mice treated with UA. However, *T. gondii*-infected mice treated with 100 μg/mL of UA showed that the effect of UA did not consistently maintain the survival of the infected mice by decreasing after 11.8 days. This result suggests that the death of mice may be owing to tissue damage and cytolysis by invasion of *T. gondii* than the loss of body weight of mice. Furthermore, symptoms and side effects, such as the loss of body weight, were not observed or induced in all of the groups treated with 200 and 400 μg/mL of UA for the experimental periods (Figure 3). The result shows effective and clear evidence regarding the effect of anti-*T. gondii* compounds, which indicates clarity regarding the results as a fusion method for both “in vitro” and “in vivo”. These results demonstrate that UA has the selective anti-parasitic activity which strongly causes anti-*T. gondii* effects as well as suppressing their viability by effectively inhibiting *T. gondii*. 

## 3. Discussion 

Until recently, the efforts for the treatment of neglected infectious diseases have been attempted at various fields. In particular, the global supporting organizations, including WHO, Bill & Melinda Gates Foundation, and the global pharmaceuticals are supporting the study for developing novel drugs and for effectively treating or blocking zoonosis such as malaria and tuberculosis, as well as infectious diseases, including AIDS, Zika, Ebola, MERS, and SARS. We should carefully focus on the neglected infectious diseases that are consistently caused through various pathways and infection factors. From these perspectives, *T. gondii* causes to fetus serious infectious diseases such as retinochoroiditis, hydrocephalus, and cerebral calcification through fetus infection during pregnancy, as well as induces symptoms, such as lymphadenopathy and meningoencephalitis in brain through acquired infection as a zoonotic parasite that is infected in both human and animals. It also induces serious complications in immune deficient patients and HIV patients as one of the infectious parasites that induce parasitic zoonosis, which may induce the interaction for parasitic proliferation and growth through parasitic relationship such as symbiosis in the host. In this aspect, *T. gondii* not only inhibits defensive mechanisms of cytokines, such as IL-2, -4, -6, -8, and IFN-γ released from host cells after invasion, but also suppresses the production of protective systems that are activated through the immune-response. Furthermore, it is known to block the signaling transport pathways that activate anti-parasitic system and the resistance to *T. gondii* invasion in host cells, as well as to suppress the apoptotic signals and cell arrest pathways during the early apoptotic stage of host cells after cell invasion [32,33]. Moreover, *T. gondii* promotes inactivation of cell cycle initiators and apoptotic mediators during the proliferative phase of *T. gondii* by forming PVM of *T. gondii* in host cells, which rapidly accelerates the proliferation of *T. gondii* in PVM [34].

In this study, we evaluated the anti-parasitic activity/effect of UA which inhibits the proliferation of *T. gondii*, and confirmed the viability of *T. gondii* after the parasite infection through *T. gondii*-infected in vitro system and animal testing. In the present study, the expression of PVM in the infected host cells was significantly increased when compared with *T. gondii*-infected cells treated with UA and SF, and uninfected cells. On the other hand, the proliferation of *T. gondii* and PVM were markedly inhibited in *T. gondii*-infected cells treated with UA compared with *T. gondii*-infected cells. In addition, we evaluated the anti-*T. gondii* activity of UA through *T. gondii*-infected mice. After the injection of *T. gondii* treated with UA (200 and 400 μg/mL), the mice showed the vitality and the viability such as the uninfected control group without the loss of body weight and the cytotoxicity during the experiment. In particular, it was recently reported that UA has the anti-protozoa effects against *Leishmania amazonesis* and *Leishmania infantums*, causing leishmaniasis among the neglected tropical diseases, and its mechanism of action is associated with programmed cell death and nitric oxide (NO) production [35,36]. In this aspect, the anti-*T. gondii* effect of UA suggests that UA may induce the anti-proliferation and the growth inhibitory action of *T. gondii* through the mechanism of action that is caused by nitric oxide (NO) production or programmed cell death in *T. gondii* and the infected host cells.

Taken together, these results indicate that the expression of PVM in *T. gondii*-infected cells was selectively inhibited or blocked in the host cells treated with UA, as well as significantly increased in infected cells. Furthermore, the *T. gondii*-infected mice treated with UA showed a stable life-cycle and survival rates when compared with infected positive group during the experimental period. The results clearly provide the inhibitory effect and activity of the compound against *T. gondii*, and indicate whether UA induces the direct inhibition of *T. gondii* or increase the survival of mice by effectively blocking and inhibiting the viability of *T. gondii*. 

## 4. Materials and Methods 

### 4.1. Materials 

Fetal bovine serum (FBS), antibiotics, and trypsin-EDTA were purchased from Invitrogen Corporation (Gibco^®^, Waltham, MA, USA). MTT (3-(4,5-dimethylthiazol-2-yl)-2,5-diohenyl-2H-tetrazolium bromide; Thiazolyl blue), RPMI medium 1640, sulfadiazine, dimethyl sulfoxide (DMSO), phosphate buffered saline (PBS), 0.4% trypan blue solution, and Hoechst 33342 were purchased from Sigma-Aldrich Chemical Co., Ltd. (St. Louis, MO, USA). All the other chemicals and reagents were purchased from Merck Chemical Co., Ltd. (Darmstadt, Germany) and Sigma-Aldrich Chemical Co., Ltd. (St. Louis, MO, USA). 

### 4.2. Animals 

BALB-c/mice (six weeks, *n* = 25) were purchased from DaeHan Bio-Link Co., Ltd. (Chungcheongbuk-do, Korea), and all animals were kept at 23 ± 0.5 °C and 12 h-light/dark cycle in a controlled environment of a central animal care facility. Food and water were provided ad libitum to all animals. The facility was strictly maintained in accordance with the guidelines of the National Institutes of Health for the Care and Use of Laboratory Animals. 

### 4.3. Preparation of Anti-T. gondii Drugs 

The anti-*T. gondii* drug, sulfadiazine (SF), was dissolved in DMSO and ursolic acid (UA) was also dissolved in DMSO to a concentration of 50 mg/mL, according to the manufacturer’s instruction. Sulfadiazine was used as a standard drug to evaluate whether or not ursolic acid has an anti-parasitic effect and activity against *T. gondii*. All of the compounds were filtered using 0.2 µm membrane syringe filters (Roshi Kaisha, Ltd., Tokyo, Japan) before use, and were stored at −80 °C deep-freezer until use. 

### 4.4. Cell Lines and Culture Conditions of T. gondii 

Glioma cells (C6 cells) were purchased from Korean Cell Line Bank at Seoul National University. C6 cells were cultured in RPMI medium 1640 containing 2 mM l-glutamine, supplemented with 10% fetal bovine serum (FBS), 100 units/mL penicillin, and 100 μg/mL streptomycin (Biofluids, Rockville, MD, USA) in a humidified atmosphere containing 5% CO_2_ in air at 37 °C. The RH strain of *T. gondii* was suspended with 1X PBS, which was injected in the abdominal cavity of each BALB-c/mouse. Five days after the injection, *T. gondii* was collected from the peritoneal fluids of each mouse kept in the abdominal cavities of the mice before use in the studies. In the in vitro study, cells were infected with *T. gondii* (cells:*T. gondii* = 1:5). 

### 4.5. Evaluation of The Viability of T. gondii 

To evaluate the inhibitory effects of UA against the viability of *T. gondii*, we investigated the viability of *T. gondii* exposed to UA and SF. Briefly, after *T. gondii* was seeded in a 24 well plate (1 × 10^7^/well), *T. gondii* was incubated with different concentrations (50–200 μg/mL) of UA and SF for 24 h, respectively, and their viabilities were determined by MTT assay. It is to determine whether or not UA has direct anti-parasitic activity and/or effect against *T. gondii*. The survival rate (SR) of *T. gondii* was calculated, as follows: % of SR = (OD_drug-tested wells_ − OD_blank_)/(OD_control_ − OD_blank_) × 100. The optical density (OD) was measured at a wavelength of 570 nm using an ELISA leader. 

### 4.6. Microscopic Observation of T. gondii in Infected Cells 

The cells were seeded in a 24 well plate (1 × 10^5^/well), which were incubated at 37 °C for 24 h. The cells were infected with *T. gondii* (5 × 10^5^ tachyzoites/well), and then *T. gondii*-infected cells were treated with 100 μg/mL of UA and SF for 24 h, respectively. The morphological changes of *T. gondii*-infected host cells were observed under a light microscope (Nikon Eclipse TE 2000-U, Tokyo, Japan). 

### 4.7. Nuclear Staining of T. gondii-Infected Host Cells 

The anti-*T. gondii* effect of UA against *T. gondii*-infected cells was evaluated using Hoechst 33342, according to the nuclear staining method described previously [37]. The cells were seeded onto cover slips in a 24 well plate (1 × 10^5^ cells/well), which were infected with *T. gondii* (5 × 10^5^ tachyzoites/well) after 24 h. The *T. gondii*-infected cells were incubated with 100 μg/mL of UA and SF for 24 h respectively. After washing with 1X PBS, the *T. gondii*-infected cells were fixed in 1X PBS containing 5% formaldehyde for 30 min, and then washed with 1X PBS and stained with a final concentration of 20 μM (Hoechst 33342, St. Louis, MO, USA) for 30 min in the dark. After nuclear staining, the cells were washed with 1X PBS three times, and their nuclear changes were observed under a UV fluorescent microscope (Nikon Eclipse TE 2000-U, Tokyo, Japan). 

### 4.8. PVM Formation in T. gondii-Infected Cells 

The cells were seeded onto cover slips in a 24 well plate (1 × 10^5^ cells/well), which were infected with *T. gondii* (5 × 10^5^ tachyzoites/well). *T. gondii*-infected cells were incubated with 100 μg/mL of UA and SF for 24 h, respectively. After washing with 1X PBS, they were fixed with 5% formaldehyde for 10 min and 0.05% (*v*/*v*) Triton X-100 for 5 min. The cells were blocked with 1X PBS containing 1% BSA for 1 h at room temperature after washing. A mouse monoclonal anti-PVM antibody was diluted with 1:100 (*v*/*v*) using 1% BSA/PBS, and then the cells were incubated with anti-PVM antibody solution at room temperature for 1 h. After washing, goat anti-mouse IgG-FITC-conjugated secondary antibody was diluted with 1:100 (*v*/*v*) using 1X PBS, which was added to each well. The cells were incubated at room temperature for 1 h, and were washed with 1X PBS every 15 min, four times. Their fluorescence was observed under a UV fluorescent microscope (Nikon Eclipse TE 2000-U, Tokyo, Japan). 

### 4.9. The Survival Rate of T. gondii-Infected Mice 

Twenty-five animals (mouse/6 weeks, *n* = 25) were divided into normal (*n* = 5) and experimental groups (four groups, *n* = 20). *T. gondii* was seeded in a 12 well plate (4 × 10^6^/well), which was incubated with different concentrations (100, 200, and 400 μg/mL) of UA for 24 h, respectively. *T. gondii* treated with the compounds was harvested before the injection in the abdominal cavity of each mouse in the experiment group, which was washed with 1X PBS three times. The pellets were suspended with 1X PBS, which were injected in the abdominal cavity of each BALB-c/mouse in the experimental groups. *T. gondii*-infected mice untreated with UA were used as an infected positive group, and the animals were kept in a central animal care facility during the experiment. 

### 4.10. Statistical Analysis 

All of the results were expressed as mean ± S.D. Statistical analysis of the data was performed using Student’s *t*-test and analysis of variance (ANOVA). * *p* < 0.05 was considered to be statistically significant. 

## 5. Conclusions

The results of this study demonstrate that UA not only has anti-*T. gondii* activity causing the direct inhibition of *T. gondii,* but also causes anti-parasitic effect against *T. gondii* by strongly inhibiting the proliferation and growth of *T. gondii* in infected host cells. In addition, our results show clearly for the first time that UA has anti-*T. gondii* effect which consistently induces the survival of *T. gondii*-infected mice. Therefore, this study provides substantial results and the potential that UA can be utilized as a promising candidate substance for anti-*T. gondii* drug development of next-generation through *T. gondii*-infected cells and infected-mice model. In addition, UA suggests novel perspectives of the approach for anti-*T. gondii* drug development, which shows the need of further study regarding the safety and/or efficacy against toxoplasmosis through preclinical study in the near future.

## Figures and Tables

**Table 1:** The 50% inhibitory concentration value of ursolic acid against the viability of *T. gondii* and *T. gondii*–infected cells measured by the MTT assay.

The Tested Compound	Structure (C_30_H_48_O_3)_	The IC_50_ (µg/mL) of Ursolic Acid against *T. gondii*	The IC_50_ (µg/mL) of Ursolic Acid against *T. gondii*–Infected Cells
Ursolic acid (UA)	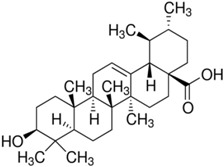	94.62	162.25

The anti-*T. gondii* effect of ursolic acid against the proliferation and the viability of *T. gondii* and *T. gondii*–infected cells was measured using the MTT assay. They were incubated with different concentrations (25–200 µg/mL) of the compound at 37 °C for 24 h respectively. IC_50_ (50% inhibitory concentration value of UA against the survival rate of *T. gondii* and *T. gondii*–infected cells). The results were carried out three times independently.

**Table 2:** The inhibitory effect of ursolic acid against the growth and the proliferation of *T. gondii*-infected cells.

Infection Ratio of *T. gondii* (MOI)	Incubation Time	Concentrations (µg/mL)	The Survival Rate (%) of *T. gondii*-Infected Cells	
			Ursolic Acid (UA)	Sulfadiazine (SF)
Cells:*T. gondii* = 1:5	24 h	0	100.00 ± 2.28	100.00 ± 3.46
		25	92.35 ± 2.74	94.26 ± 1.82
		50	85.52 ± 1.55	91.35 ± 1.64
		100	67.61 ± 1.87	77.58 ± 3.45
		200	38.42 ± 3.12	60.45 ± 1.68

*T. gondii*-infected cells were incubated with different concentrations (25–200 μg/mL) of ursolic acid (UA) and sulfadiazine (SF) for 24 h respectively, and their survival rates were measured using the MTT assay. The results were expressed as a percentage of the normal group, and all the results were presented as mean ± standard deviation (S.D.) of three independent experiments.

## References

[1] Joiner K.A., Roos D.S. (2002). Secretory traffic in the eukaryotic parasite *Toxoplasma gondii*: Less is more. J. Cell Biol..

[2] Bonhomme A., Bouchot A., Pezzella N., Gomez J., Le M.H., Pinon J.M. (1999). Signaling during the invasion of host cells by *Toxoplasma gondii*. FEMS Microbiol. Rev..

[3] Meneceur P., Bouldouyre M.A., Aubert D., Villena I., Menotti J., Sauvage V., Garin J.F., Derouin F. (2008). In vitro susceptibility of various genotypic strains of Toxoplasma gondii to pyrimethamine, sulfadiazine, and atovaquone. Antimicrob. Agents Chemother..

[4] Doliwa C., Xia D., Escotte-Binet S., Newsham E.L., Sanya J.S., Aubert D., Randle N., Wastling J.M., Villena I. (2013). Identification of differentially expressed proteins in sulfadiazine resistant and sensitive strains of Toxoplasma gondii using difference-gel electrophoresis (DIGE). Int. J. Parasitol. Drugs Drug Resist..

[5] Aspinall T.V., Joynson D.H., Guy E., Hyde J.E., Sims P.F. (2002). The molecular basis of sulfonamide resistance in Toxoplasma gondii and implications for the clinical management of toxoplasmosis. J. Infect. Dis..

[6] Choi W.H., Lee I.A. (2016). The anti-tubercular activity of *Melia azedarach* L. and *Lobelia chinensis* Lour. and their potential as effective anti-*Mycobacterium tuberculosis* candidate agents. Asian Pac. J. Trop. Biomed..

[7] Qiu L., Chen K.P. (2013). Anti-HBV agents derived from botanical origin. Fitoterapia.

[8] Wycoff K., Maclean J., Belle A., Yu L., Tran Y., Roy C., Hayden F. (2015). Anti-infective immunoadhesins from plants. Plant Biotechnol. J..

[9] De Oliveira L.H.G., Silva de Sousa P.A.P., Hilario F.F., Nascimento G.J., Morais J.P.S., de Medeiros E.P., de Sousa M.F., da Cruz Nunes F. (2016). Agave sisalana extract induces cell death in Aedes aegypti hemocytes increasing nitric oxide production. Asian. Pac. J. Trop. Biomed..

[10] Wang M., Tao L., Xu H. (2016). Chinese herbal medicines as a source of molecules with anti-enterovirus 71 activity. Chin. Med..

[11] Choi W.H. (2017). Novel pharmacological activity of artesunate and artemisinin: Their potential as anti-tubercular agents. J. Clin. Med..

[12] Kavitha N., Noordin R., Chan K.L., Sasidharan S. (2012). In vitro anti-*Toxoplasma gondii* activity of root extract/fractions of Eurycoma longifolia Jack. BMC Complement. Altern. Med..

[13] Alomar M.L., Rasse-Suriani F.O., Ganuza A., Coceres V.M., Cabrerizo F.M., Angel S.O. (2013). In vitro evaluation of b-carboline alkaloids as potential anti-Toxoplasma agents. BMC Res. Notes.

[14] Dzitko K., Grzybowski M.M., Pawełczyk J., Dziadek B., Gatkowska J., Sta˛czek P., Długonska H. (2015). Phytoecdysteroids as modulators of the *Toxoplasma gondii* growth rate in human and mouse cells. Parasites Vectors.

[15] Gasparotto Junior A., Cosmo M.L., Reis Mde P., Dos Santos P.S., Gonçalves D.D., Gasparotto F.M., Navarro I.T., Lourenço E.L. (2016). Effects of extracts from *Echinacea purpurea* (L.) MOENCH on mice infected with different strains of *Toxoplasma gondii*. Parasitol. Res..

[16] Zhang X., Jin L., Cui Z., Zhang C., Wu X., Park H., Quan H., Jin C. (2016). Antiparasitic effects of oxymatrine and matrine against *Toxoplasma gondii* in vitro and in vivo. Exp. Parasitol..

[17] Colla A.R., Rosa J.M., Cunha M.P., Rodrigues A.L. (2015). Anxiolytic-like effects of ursolic acid in mice. Eur. J. Pharmacol..

[18] Kanjoormana M., Kuttan G. (2010). Antiangiogenic activity of ursolic acid. Integr. Cancer Ther..

[19] Kazmi I., Afzal M., Gupta G., Anwar F. (2012). Antiepileptic potential of ursolic acid stearoyl glucoside by GABA receptor stimulation. CNS Neurosci. Ther..

[20] Lu J., Wu D.M., Zheng Y.L., Hu B., Zhang Z.F., Ye Q., Liu C.M., Shan Q., Wang Y.J. (2010). Ursolic acid attenuates d-galactose-induced inflammatory response in mouse prefrontal cortex through inhibiting AGEs/RAGE/NF-κB pathway activation. Cereb. Cortex.

[21] Checker R., Sandur S.K., Sharma D., Patwardhan R.S., Jayakumar S., Kohli V., Sethi G., Aggarwal B.B., Sainis K.B. (2012). Potent anti-inflammatory activity of ursolic acid, a triterpenoid antioxidant, is mediated through suppression of NF-κB, AP-1 and NF-AT. PLoS ONE.

[22] Zhang Y.X., Kong C.Z., Wang L.H., Li J.Y., Liu X.K., Xu B., Xu C.L., Sun Y.H. (2010). Ursolic acid overcomes Bcl-2-mediated resistance to apoptosis in prostate cancer cells involving activation of JNK-Induced Bcl-2 phosphorylation and degradation. J. Cell. Biochem..

[23] Choi W.H., Chu J.P., Jiang M.H., Baek S.H., Park H.D. (2011). Effects of fraction obtained from Korean Corni Fructus extracts causing anti-proliferation and p53-dependent apoptosis in A549 lung cancer cells. Nutr. Cancer.

[24] Prasad S., Yadav V.R., Sung B., Gupta S.C., Tyagi A.K., Aggarwal B.B. (2016). Ursolic acid inhibits the growth of human pancreatic cancer and enhances the antitumor potential of gemcitabine in an orthotopic mouse model through suppression of the inflammatory microenvironment. Oncotarget.

[25] Ramos A.A., Pereira-Wilson C., Collins A.R. (2010). Protective effects of ursolic acid and luteolin against oxidative DNA damage include enhancement of DNA repair in Caco-2 cells. Mutat. Res..

[26] Do Nascimento P.G., Lemos T.L., Bizerra A.M., Arriaga A.M., Ferreira D.A., Santiago G.M., Braz-Filho R., Costa J.G. (2014). Antibacterial and antioxidant activities of ursolic acid and derivatives. Molecules.

[27] Jimenez-Arellanes A., Luna-Herrera J., Cornejo-Garrido J., Lopez-Garcia S., Castro-Mussot M.E., Meckes-Fischer M., Mata-Espinosa D., Marquina B., Torres J., Hernández-Pando R. (2013). Ursolic and oleanolic acids as antimicrobial and immunomodulatory compounds for tuberculosis treatment. BMC Complement. Altern. Med..

[28] Kang S.Y., Yoon S.Y., Roh D.H., Jeon M.J., Seo H.S., Uh D.K., Kwon Y.B., Kim H.W., Han H.J., Lee H.J. (2008). The anti-arthritic effect of ursolic acid on zymosan-induced acute inflammation and adjuvant-induced chronic arthritis models. J. Pharm. Pharmacol..

[29] Xu H., Zhang M., Li X.L., Li H., Yue L.T., Zhang X.X., Wang C.C., Wang S., Duan R.S. (2015). Low and high doses of ursolic acid ameliorate experimental autoimmune myasthenia gravis through different pathways. J. Neuroimmunol..

[30] Borges I.P., Castanheira L.E., Barbosa B.F., de Souza D.L., da Silva R.J., Mineo J.R., Tudini K.A., Rodrigues R.S., Ferro E.A., de Melo R.V. (2016). Anti-parasitic effect on *Toxoplasma gondii* induced by BnSP-7, a Lys49-phospholipase A2 homologue from Bothrops pauloensis venom. Toxicon.

[31] Sanfelice R.A., da Silva S.S., Bosqui L.R., Miranda-Sapla M.M., Barbosa B.F., Silva R.J., Ferro E.A.V., Panagio L.A., Navarro I.T., Bordignon J. (2017). Pravastatin and simvastatin inhibit the adhesion, replication and proliferation of Toxoplasma gondii (RH strain) in HeLa cells. Acta Trop..

[32] Carmen J.C., Southard R.C., Sinai A.P. (2008). The complexity of signaling in host-pathogen interactions revealed by the Toxoplasma gondii-dependent modulation of JNK phosphorylation. Exp. Cell Res..

[33] Laliberte J., Carruthers V.B. (2008). Host cell manipulation by human pathogen *Toxoplasma gondii*. Cell. Mol. Life Sci..

[34] Gubbels M.J., White M., Szatanek T. (2008). The cell cycle and *Toxoplasma gondii* cell division: Tightly knit or loosely stitched. Int. J. Parasitol..

[35] Jesus J.A., Fragoso T.N., Yamamoto E.S., Laurenti M.D., Silva M.S., Ferreira A.F., Lago J.H., Santos-Gomes G., Passero L.F. (2017). Therapeutic effect of ursolic acid in experimental visceral leishmaniasis. Int. J. Parasitol. Drugs Drug Resist..

[36] Yamamoto E.S., Campos B.L., Jesus J.A., Laurenti M.D., Ribeiro S.P., Kallas E.G., Rafael-Fernandes M., Santos-Gomes G., Silva M.S., Sessa D.P. (2015). The Effect of Ursolic Acid on *Leishmania* (*Leishmania*) *amazonensis* is Related to Programed Cell Death and Presents Therapeutic Potential in Experimental Cutaneous Leishmaniasis. PLoS ONE.

[37] Latt S.A., Stetten G. (1976). Spectral studies on 33258 Hoechst and related bisbenzimidazole dyes useful for fluorescent detection of deoxyribonucleic acid synthesis. J. Histochem. Cytochem..

